# Serum tRF-4575 may regulate osteoclast differentiation and serve as a promising biomarker for enthesitis-related arthritis diagnosis

**DOI:** 10.1016/j.gendis.2025.101848

**Published:** 2025-09-10

**Authors:** Jing Jin, Lingzhi Qiu, Yuting Pan, Yifan Xie, Xiaoyan Shao, Zhidan Fan, Haiguo Yu

**Affiliations:** Department of Rheumatology and Immunology, Children's Hospital of Nanjing Medical University, Nanjing, Jiangsu 210008, China

Patients with enthesitis-related arthritis (ERA) exhibit limited spinal function and clinically observable sacroiliitis,^1^ and they are predisposed to experiencing higher disease activity, reduced rates of disease remission, and poorer long-term outcomes compared with other subtypes of juvenile idiopathic arthritis. Nevertheless, due to the insidious onset and the subtle clinical manifestations during the early stages of the disease, achieving a timely diagnosis remains a significant challenge for children affected by ERA.^2^^,^^3^ Consequently, there is a pressing necessity to develop innovative, noninvasive diagnostic biomarkers with high efficacy for the screening of children with ERA. Transfer RNA (tRNA)-derived small RNAs (tsRNAs) or tRNA-derived fragments (tRFs), which are abundantly present in serum, have been reported to participate in fundamental regulatory processes and exhibit a strong association with various diseases.^4^ Recent research suggests that tRFs hold potential as diagnostic biomarkers and may be valuable for monitoring disease progression and determining disease prognosis.^4^^,^^5^ However, data on tRFs in rheumatoid arthritis and ERA remain scarce. Therefore, this study aims to investigate the expression profile of tRFs in children with ERA and assess their potential diagnostic value by examining the distribution of these small non-coding RNAs in ERA serum samples. Additionally, preliminary investigations into related biological functions were conducted using bioinformatics approaches.

To examine the expression profiles of tRFs in the serum of patients with ERA and normal controls (NCs), small RNA sequencing was conducted on serum samples from five ERA patients and five NCs (Table S1). Clustering analyses identified significantly altered expression patterns of tRFs within these samples (Fig. 1A). The scatter plot demonstrated that under the criteria of fold change ≥2 and *p* < 0.05, a total of 161 tRFs exhibited significantly differential expression, with 98 being up-regulated and 63 down-regulated (Fig. 1B). These tRFs were further classified into various subtypes based on their size and length. Figure 1C presents a pie chart depicting the distribution and quantity of each subtype in both groups, highlighting a more pronounced difference in 3′-tRF. These findings indicate that the expression profiles of tRFs differ markedly in the serum samples of children with ERA compared with those in the serum of NCs.

**Figure 1:**
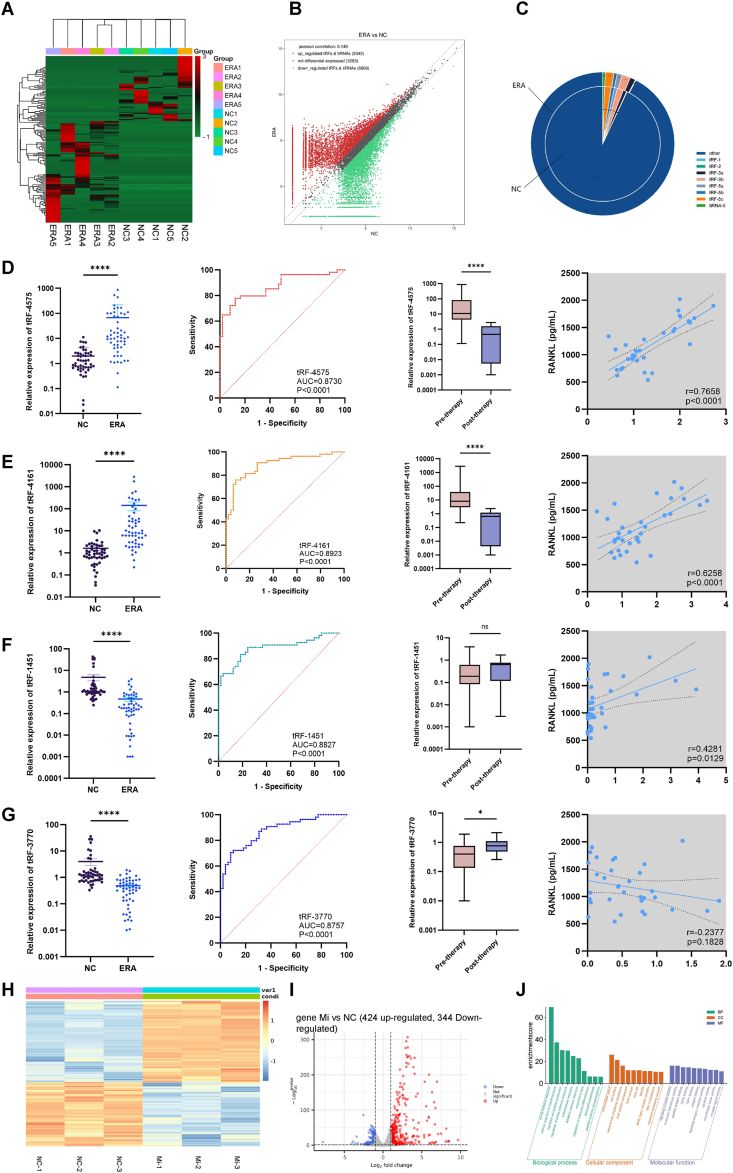
Serum tRF profiles and functional characterization of tRF-4575 in children with ERA. **(A)** Heatmap of hierarchical clustering of differentially expressed tRFs in serum samples from five ERA patients and five NCs, with red indicating high expression and green indicating low expression. **(B)** The scatter plot illustrating the comparison of tRFs between ERA and NCs. **(C)** The pie chart depicting the distribution and quantity of tRF subtypes. **(D**–**G)** Relative expression levels, receiver operating characteristic (ROC) curves, expression changes pre- and post-treatment, and correlation with RANKL levels for tRF-4575, tRF-4161, tRF-1451, and tRF-3770. **(H)** Heatmap of differentially expressed genes (DEGs) in RAW264.7 cells transfected with the tRF-4575 mimic and mimic-NC, with red and blue indicating up- and down-regulated genes, respectively. Mi represents the mimic group, and NC represents the mimic-NC group. **(I)** Volcano plot of DEGs between tRF-4575 mimic and mimic-NC. Mi represents the mimic group, and NC represents the mimic-NC group. **(J)** Gene Ontology (GO) analysis for the potential pathways of DEGs.

To further verify the expression levels of tRFs in patients with ERA (Table S2), two significantly up-regulated tRFs (tRF-4575 and tRF-4161) and two significantly down-regulated tRFs (tRF-1451 and tRF-3770) were selected for analysis (Table S3). Quantitative reverse transcription PCR was employed to validate the expression of those four candidate tRFs. Consistent with the initial sequencing results, both tRF-4575 (Fig. 1D) and tRF-4161 (Fig. 1E) exhibited significant up-regulation in patients with ERA (*p* < 0.001). Conversely, the expression levels of tRF-1451 (Fig. 1F) and tRF-3770 (Fig. 1G) were significantly lower in the experimental group compared with the NC group (*p* < 0.001).

Given the substantial variation in the relative levels of the selected tRFs, we conducted additional analyses to further investigate their potential. To evaluate the clinical utility of these tRFs, we employed receiver operating characteristic (ROC) curves to determine their predictive sensitivity and specificity. As illustrated in Figure 1D–G, the area under the curve (AUC) values for tRF-4575, tRF-4161, tRF-1451, and tRF-3770 were 0.8730 (95% confidence interval/CI: 0.8046–0.9414; *p* < 0.0001), 0.8923 (95% CI: 0.8046–0.9414; *p* < 0.0001), 0.8827 (95% CI: 0.8153–0.9500; *p* < 0.0001), and 0.8757 (95% CI: 0.8104–0.9410; *p* < 0.0001), respectively. These findings indicate that all four tRFs exhibit high diagnostic specificity and accuracy.

Subsequently, we evaluated the alterations in the expression of selected tRFs following clinical treatment using quantitative reverse transcription PCR. Our analysis revealed that the expression levels of tRF-4575 (Fig. 1D) and tRF-4161 (Fig. 1E) before treatment were significantly elevated compared with those post-treatment. This suggests that tRF-4575 and tRF-4161 may serve as reliable prognostic markers for ERA. Furthermore, the serum level of tRF-3770 in patients showed a significant increase with therapy (*p* = 0.0231) (Fig. 1G), whereas no significant change was observed in the expression of tRF-1451 before and after treatment (*p* = 0.2113) (Fig. 1F).

In addition, we assessed the correlation between the receptor activator of nuclear factor-κB (RANK) ligand (RANKL) levels and the expression of the selected tRFs in serum. Our analysis revealed that the expression of tRF-4575 (Fig. 1D), tRF-4161 (Fig. 1E), and tRF-1451 (Fig. 1F) exhibited a positive correlation with serum RANKL levels, with tRF-4575 showing a particularly strong association. Conversely, no correlation was observed between tRF-3770 and RANKL (Fig. 1G). Given the critical role of RANKL in osteoclast differentiation and subsequent bone resorption in patients with ERA, these results suggest that tRF-4575, tRF-4161, and tRF-1451 may be implicated in bone metabolism and contribute to the pathogenesis of ERA.

Based on the observed significant positive correlation between tRF-4575 and RANKL levels, we further investigated the molecular role of tRF-4575 in osteoclast differentiation through next-generation sequencing (Fig. 1H). Specifically, we conducted a comparative analysis between the tRF-4575 mimic and mimic-NC, which revealed 768 differentially expressed mRNA transcripts (|log_2_ fold change| ≥ 1; *p* < 0.05). Of these, 424 transcripts were down-regulated, while 344 were up-regulated in the NC group (Fig. 1I). To elucidate the potential functions of tRF-4575, we performed Gene Ontology (GO) (Fig. 1J; Fig. S1A–C) and Kyoto Encyclopedia of Genes and Genomes (KEGG) (Fig. S1D and E) analyses on the associated target genes. As shown in the GO analysis, the target genes of tRF-4575 are predominantly involved in immune and inflammatory responses, cytokine production regulation, osteoclast differentiation, and signaling receptor activity. Meanwhile, KEGG analysis revealed significant enrichment of tRF-4575 target genes in pathways related to antigen processing and presentation, various inflammatory signaling pathways, rheumatoid arthritis, and osteoclast differentiation. The convergence observed in the GO and KEGG analyses of these tRF-regulated genes involved in osteoclast differentiation aligns with their association with RANKL levels. This supports our hypothesis that tRF-4575 may promote osteoclast differentiation, exacerbate bone resorption, and contribute to disease progression. In light of the bioinformatics analysis findings, conducting both *in vivo* and *in vitro* experiments to validate the regulatory role of tRF-4575 in bone metabolism within the context of juvenile idiopathic arthritis or ERA is a crucial direction for future research.

In conclusion, this study analyzed serum tRF expression profiles in pediatric patients with ERA, identifying tRF-4575 as a promising, sensitive, and novel biomarker for the diagnosis and prognosis of ERA. Bioinformatic analysis further suggested that tRF-4575 may play a role in the pathogenesis of ERA by influencing osteoclast differentiation. These findings contribute a new theoretical framework for understanding the pathogenesis of ERA and may present a potential therapeutic target for its treatment.

## CRediT authorship contribution statement

**Jing Jin:** Investigation, Writing – original draft. **Lingzhi Qiu:** Data curation. **Yuting Pan:** Data curation. **Yifan Xie:** Writing – review & editing. **Xiaoyan Shao:** Writing – review & editing. **Zhidan Fan:** Conceptualization, Methodology. **Haiguo Yu:** Methodology.

## Ethics declaration

The study protocol was approved by the Ethics Committee of the Children's Hospital Affiliated with Nanjing Medical University (No. 202008041-1). Simultaneously, written informed consent was obtained from all participants' parents or guardians.

## Data availability

The datasets generated during and analyzed during the current study are not publicly available due to privacy and ethical restrictions but are available from the corresponding author upon reasonable request.

## Funding

This study was supported by the 10.13039/501100001809National Natural Science Foundation of China (No. 82271838) and the Project of the 10.13039/100017962Jiangsu Provincial Health Commission (No. M2021080, M2022018).

## Conflict of interests

The authors declared no conflict of interests.
