# Supplementary material for: Serum tRF-4575 may regulate osteoclast differentiation and serve as a promising biomarker for enthesitis-related arthritis diagnosis
Source: Genes Dis. 2025 Sep 10;13(3):101848. doi: 10.1016/j.gendis.2025.101848 (PMC12854862; doi:10.1016/j.gendis.2025.101848)
Supplement: Multimedia component 1 [file mmc1.docx]

**Materials and methods**

**Clinical samples collection**

This study included 59 ERA patients and 54 healthy children hospitalized at the Children’s Hospital Affiliated with Nanjing Medical University, from February 2022 to July of 2024. Five new-onset ERA patients and five healthy controls (HCs) were selected randomly for the primary small RNA profiling analysis and the additional 54 ERA patients and 49 HCs were enrolled as validation cohort. Their clinical data are listed in Table 1 and Table 2. All patients fulfilled the classification criteria for ERA. Samples from these participants were collected with a coagulation-promoting tube and centrifuged for 10 min at 3500 rpm at room temperature within 1 h of collection. The gained serum samples were then centrifuged at 13,000 rpm at 4 ◦C  and transferred into a 1.5 mL RNase-free polypropylene (PP) tube. All collected samples were stored at −80 ◦C until RNA isolation within three months for the following experiments.

The study protocol was approved by the Ethics Committee of the Children’s Hospital Affiliated with Nanjing Medical University (No.202008041-1) and written informed consent was obtained from all participants’ parents or guardians.

**tRFs and tiRNA sequencing**

Firstly, the integrity and quantity of each RNA sample were assessed with agarose gel electrophoresis and a NanodropTM instrument (Thermo Fisher Scientific, USA) and the results showed that tRFs are heavily decorated with RNA modifications. So, the total samples were pretreated with multiple steps to remove those decoration. The pretreated total RNA samples were then subjected to library preparation using an Agilent BioAnalyzer 2100 (Agilent Technologies, USA). The completed libraries were sequenced with 50-bp single-read on the NextSeq instrument (Illumina, USA). The sequencing quality was assessed by FastQC software. Next, the trimmed reads were compared with the tRNA precursor sequence from GtRNAdb using the Novo Align software (v2.07.11). And the other unrecognized reads were aligned to other corresponding databases (mRNA/rRNA/snRNA/snoRNA/piRNA/miRNA ). The expression level of tRFs was measured and normalized to the number of transcripts per million of total aligned tRNA reads (TPM). The expression profiles between the ERA patients and the HCs were compared by calculating the fold change of each tRF. Based on the sequencing results, we further selected 2 up-regulated tRFs and 2 down-regulated tRFs with fold change ≥ 2, P value < 0.05 and significant differences between the two groups for subsequent experiments, namely tRF-4575, tRF-4161, tRF-1451 and tRF-3770, respectively. Their sequences and parental tRNA sequences are listed in Table3.

**RNA extraction and qRT-PCR**

Total RNA from serum was isolated using TRIzol (Vazyme, China). The yield and purity of RNA were measured using NanoDrop 1000 (Thermo Fisher, USA). cDNA was generated using the Bulge-loop™ qRT-PCR Primer Sets (Ribobio, China) and HiScript II Q RT SuperMix for qPCR (Vazyme, China), following the manufacturer’s instructions. Real-time PCR was performed on an ABI QuantStudio 3 (Thermo Fisher Cloud, USA). The expression of targeted genes and tRFs were normalized to U6 or cel-miR 39-3p Standard RNA (RiboBio, China), respectively. Standard RNA and RT primer were designed and synthetized by RIBOBIO Biotech and Generay Biotech. The 2-ΔΔCt Method was taken to calculate the relative expression levels of the target genes. Three replicate experiments were conducted on each sample.

**Enzyme-linked immunosorbent assay (ELISA)**

To measure the RANKL level of ERA patients before any treatment, the collected serum samples were subjected to a RANKL ELISA kit according to the manufacturer's protocols (YOBIBIO, China).

**Cell culture and transfection**

RAW264.7 cells (ATCC, USA) were seeded at a density of 1× 10^4^ cells per well into 24-well plates and cultured in DMEM containing 10% FBS and 1% penicillin/streptomycin. For osteoclastogenic differentiation, 50 ng·mL−1 RANKL(R&D, USA) and 30 ng·mL−1 M-CSF (UA, China) were added to the cells for 5-7 days and the medium was replaced every 2 days. The mimic of tRF-4575 or negative control (RIBOBIO, China) was transfected at 100nM using lipomaster 3000 (Vazyme, China). After 48 h of incubation, cells were harvested and collected for small RNA sequencing.

**Statistical analysis**

The data are presented as the mean ± standard deviation. Two-tailed Student’s t-test or Mann–Whitney test was used to compare the differences between the two groups and Spearson’s correlation test was taken to evaluate the correlations between RANKL level and tRFs expression. Chi-square test was used to compare the gender between the two groups. All the calculations mentioned above were conducted by IBM SPSS Statistics 20.0 and the GraphPad Prism 10 software (GraphPad Software). P value less than 0.05 was chosen as the cut off for statistical significance.

**Table S1** Characteristics of ERA and HCs for sequencing.

|  | ERA(n=5) | HC(n=5) | P |
| --- | --- | --- | --- |
| Male sex | 5(100%) | 5(100%) | ＞0.05 |
| Age at onset (mean ± SD years) | 11.70±1.85 | 11.18 ± 1.52 | ＞0.05 |
| Weight at onset (mean ± SD years) | 58.90 ± 13.26 | 55.50 ± 11.61 | ＞0.05 |

**Table S2** Characteristics of ERA and HC for evaluating.

|  | ERA(n=54) | HC(n=49) | P |
| --- | --- | --- | --- |
| Male sex | 40 | 36 | ＞0.05 |
| Age at onset (mean ± SD years) | 11.36±2.20 | 11.19±1.42 | 0.643 |
| Weight at onset (mean ± SD years) | 44.43±13.99 | 47.15±8.48 | 0.24 |
| HLA-B27 positivity | 34/54 | - | - |

**Table S3** The sequences and parental tRNA sequences of the candidate tRFs

| tRFs | sequence | parental tRNA sequence |
| --- | --- | --- |
| tRF-4575 | GGTCCTGGGTTCGAGCCCCAGTGGAACCACC | GGTTCCATAGTGTAGCGGTTATCACGTCTGCTTTACACGCAGAAGGTCCTGGGTTCGAGCCCCAGTGGAACCACCA |
| tRF-4161 | CAGCGATCCGAGTTCAAATCT | GGCCCCATGGTGTAATGGTTAGCACTCTGGACTTTGAATCCAGCGATCCGAGTTCAAATCTCGGTGGGACCTCCA |
| tRF-1451 | AATGGATAAGGCATTGGCCTCCTAAGCCAGGGATTGTG | GCCCCAGTGGCCTAATGGATAAGGCATTGGCCTCCTAAGCCAGGGATTGTGGGTTCGAGTCCCATCTGGGGTGCCA |
| tRF-3770 | TTCTTGCGACCCGGGTTCGATTCCCGGGCGGCGC | GCGCCGCTGGTGTAGTGGTATCATGCAAGATTCCCATTCTTGCGACCCGGGTTCGATTCCCGGGCGGCGCACCA |
