# Supplementary material for: Serum tRF-4575 may regulate osteoclast differentiation and serve as a promising biomarker for enthesitis-related arthritis diagnosis
Source: Genes Dis. 2025 Sep 10;13(3):101848. doi: 10.1016/j.gendis.2025.101848 (PMC12854862; doi:10.1016/j.gendis.2025.101848)
Supplement: Multimedia component 2 [file mmc2.docx]

**Table S1** Characteristics of ERA and HCs for sequencing.

|  | ERA(n=5) | HC(n=5) | P |
| --- | --- | --- | --- |
| Male sex | 5(100%) | 5(100%) | ＞0.05 |
| Age at onset (mean ± SD years) | 11.70±1.85 | 11.18 ± 1.52 | ＞0.05 |
| Weight at onset (mean ± SD years) | 58.90 ± 13.26 | 55.50 ± 11.61 | ＞0.05 |

**Table S2** Characteristics of ERA and HC for evaluating.

|  | ERA(n=54) | HC(n=49) | P |
| --- | --- | --- | --- |
| Male sex | 40 | 36 | ＞0.05 |
| Age at onset (mean ± SD years) | 11.36±2.20 | 11.19±1.42 | 0.643 |
| Weight at onset (mean ± SD years) | 44.43±13.99 | 47.15±8.48 | 0.24 |
| HLA-B27 positivity | 34/54 | - | - |

**Table S3** The sequences and parental tRNA sequences of the candidate tRFs

| tRFs | sequence | parental tRNA sequence |
| --- | --- | --- |
| tRF-4575 | GGTCCTGGGTTCGAGCCCCAGTGGAACCACC | GGTTCCATAGTGTAGCGGTTATCACGTCTGCTTTACACGCAGAAGGTCCTGGGTTCGAGCCCCAGTGGAACCACCA |
| tRF-4161 | CAGCGATCCGAGTTCAAATCT | GGCCCCATGGTGTAATGGTTAGCACTCTGGACTTTGAATCCAGCGATCCGAGTTCAAATCTCGGTGGGACCTCCA |
| tRF-1451 | AATGGATAAGGCATTGGCCTCCTAAGCCAGGGATTGTG | GCCCCAGTGGCCTAATGGATAAGGCATTGGCCTCCTAAGCCAGGGATTGTGGGTTCGAGTCCCATCTGGGGTGCCA |
| tRF-3770 | TTCTTGCGACCCGGGTTCGATTCCCGGGCGGCGC | GCGCCGCTGGTGTAGTGGTATCATGCAAGATTCCCATTCTTGCGACCCGGGTTCGATTCCCGGGCGGCGCACCA |
