# Supplementary material for: Serum tRF-4575 may regulate osteoclast differentiation and serve as a promising biomarker for enthesitis-related arthritis diagnosis
Source: Genes Dis. 2025 Sep 10;13(3):101848. doi: 10.1016/j.gendis.2025.101848 (PMC12854862; doi:10.1016/j.gendis.2025.101848)
Supplement: Multimedia component 3 [file mmc3.pdf]

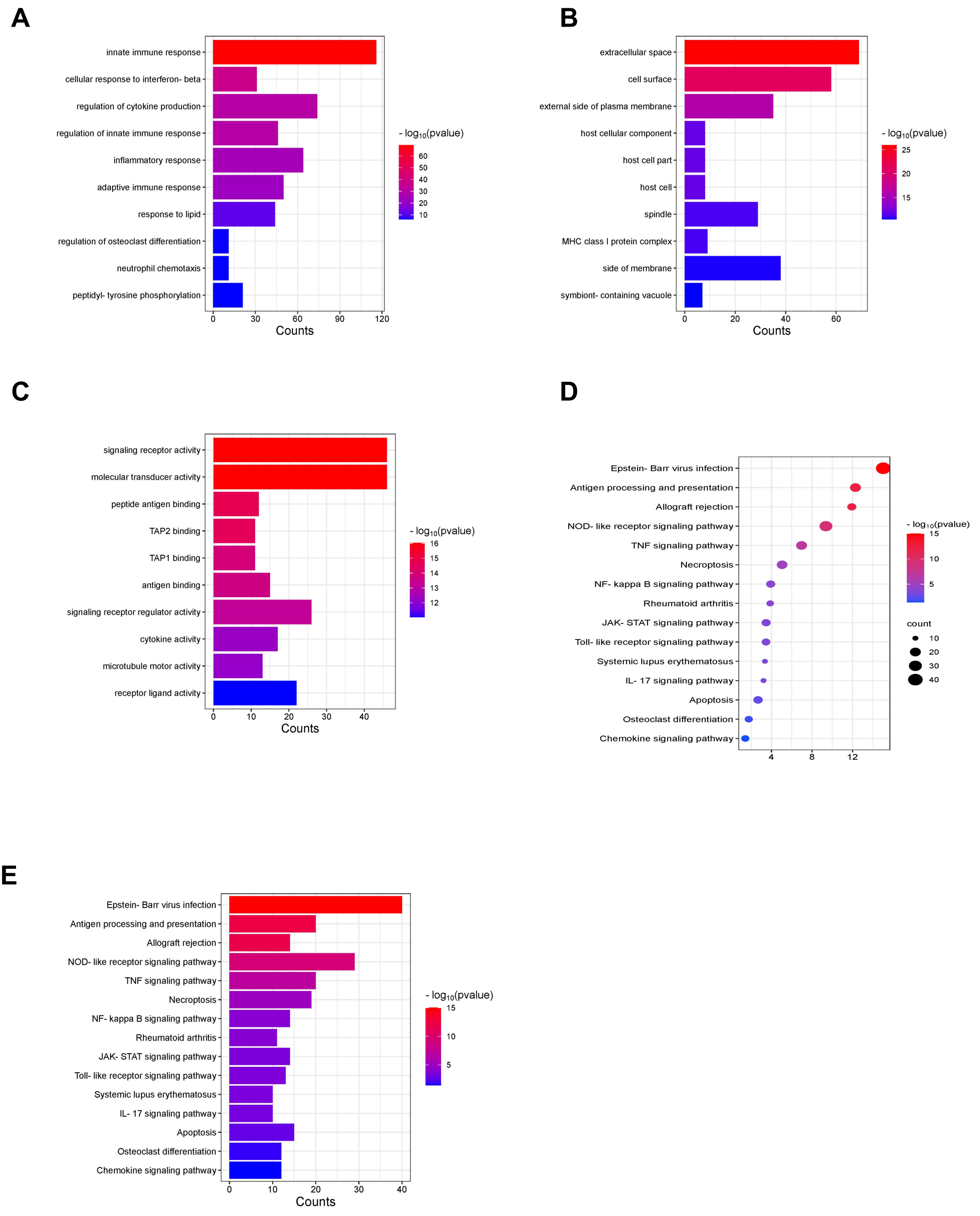

Figure S1 Bioinformatics analysis of tRF-4575-regulated genes. A-C GO analysis of DEGs based on 3 aspects(A Biological process, B Cellular component, C Molecular function). D-E KEGG pathways enrichment of DEGs.
